# Efficacy and safety of stereotactic body radiotherapy for painful bone metastases: Evidence from randomized controlled trials

**DOI:** 10.3389/fonc.2022.979201

**Published:** 2022-10-19

**Authors:** Zilan Wang, Longyuan Li, Xingyu Yang, Haiying Teng, Xiaoxiao Wu, Zhouqing Chen, Zhong Wang, Gang Chen

**Affiliations:** ^1^ Department of Neurosurgery and Brain and Nerve Research Laboratory, The First Affiliated Hospital of Soochow University, Suzhou, China; ^2^ Department of Suzhou Medical College, Soochow University, Suzhou, China

**Keywords:** bone metastases, conventional external radiation, meta-analysis, pain relief, stereotactic body radiotherapy

## Abstract

**Background:**

Pain relief is one of the main objectives of radiotherapy for cancer patients with bone metastases. Stereotactic body radiotherapy (SBRT) enables precise delivery of a higher dosage to the target area. Several trials have reported comparisons between SBRT and conventional radiotherapy (cRT) in patients with painful bone metastasis. However, the results of those investigations were inconsistent, and no systematic review or meta-analysis has been done till now.

**Methods:**

We systematically searched MEDLINE, EMBASE, Cochrane Central Register of Controlled Trials (CENTRAL), and Clinicaltrials.gov up to May 1, 2022 for relevant studies. Patients with painful bone metastasis who received SBRT or cRT were included. The primary outcome was the patients’ pain response rate at three months. The secondary outcomes included the rate of pain responders at one month and six months, oral morphine equivalent dose (OMED) use, and any adverse events. STATA software 12.0 was used for the statistical analysis.

**Results:**

We collected 533 patients’ data from 4 randomized controlled trials (RCTs), there was a significant difference of pain response rate at 3 months between two groups (RR = 1.41, 95% CI: 1.12-1.77, *I^2^* = 0.0%, P = 0.003). However, no significant difference was found in pain response rate at 1 month (RR = 1.19, 95% CI: 0.91-1.54, *I^2^* = 31.5%, P = 0.201) and 6 months (RR = 1.25, 95% CI: 0.93-1.69, *I^2^* = 0.0%, P = 0.140). OMED consumption was not significantly different in patients treated with SBRT compared with control group (WMD = -1.11, 95% CI: -17.51-15.28, *I^2^* = 0.0%, P = 0.894). For safety outcome, no statistical difference was found between SBRT and cRT (RR = 0.72, 95% CI: 0.46-1.14, *I^2^=*20.1%, P = 0.162).

**Conclusion:**

This study shows that for painful bone metastases, patients with SBRT experienced better pain relief 3 months after radiation than patients with cRT, and SBRT did not increase the incidence of adverse events.

**Systematic review registration:**

https://inplasy.com/inplasy-2022-6-0099/, identifier INPLASY202260099.

## Introduction

Bones are a common site of metastasis for some advanced cancers, such as breast and prostate cancer or lung and kidney cancer, and they can often cause several complications such as pain, pathological fractures, hypercalcemia, hemorrhage, and spinal cord compression, with sensory disturbance and dyskinesia (1). The main goal of bone metastasis therapy is to improve patients’ prognoses and prolong survival. Therefore, improving the life quality of patients is an essential topic in bone metastasis therapy, especially for pain relief. The pain can be caused by mass effect on the spinal cord or nerve roots, pathological fractures with mechanical instability, inflammation-induced periosteal nociceptor stimulation, tumor-derived products (e.g., tumor necrosis factor), or tumor-induced cytokines (2).

Some percutaneous techniques like radiofrequency ablation, cryoablation, and vertebral augmentation have been reported to be effective in alleviating pain (3). But for a long time, external beam radiotherapy (EBRT) has been considered the primary treatment for oligometastatic disease and painful bone metastases (4, 5). Previous studies have shown that conventional radiotherapy (cRT) provides pain relief in approximately 60% of patients with painful bone metastases, and complete pain relief in approximately 30% (6, 7). In recent years, new progress has been made in radiation technology. Stereotactic body radiotherapy (SBRT) has been gradually applied in radiotherapy for bone metastasis, which is based on accurate real-time assessment of tumor target location and dose delivery of multiple beam irradiation. For selected extracranial lesions (such as lung, liver, and prostate lesions), compared with cRT, its accuracy allows delivery of high doses in limited fractions (8). Studies have shown that the tumor control rate of SBRT in the treatment of bone metastases is up to 80% (9, 10). And for pain relief, SBRT also showed a complete pain relief rate of up to 50% (11).

Previously, several trials and meta-analyses focused on determining the optimal radiation dose and fractionation for painful bone metastases. While the study by Rich et al. indicated that patients who received single-fraction treatments and those who received multiple-fraction treatments had similar overall response rates (12). Several trials have reported the comparison between SBRT and cRT for survival outcomes of diseases such as non-small cell lung cancer and hepatocellular carcinoma (13, 14). However, no meta-analysis has been conducted to verify the efficacy and safety of SBRT as compared to cRT for patients with painful bone metastases. Therefore, in this study, we included randomized controlled trials (RCTs) on SBRT versus cRT in patients with painful bone metastases, and conducted the systematic review and meta-analysis to compare the effects of the two treatments.

## Methods

### Study protocol

The study was registered on the INPLASY website (INPLASY202260099) and followed the Preferred Reporting Items for Systematic Reviews and Meta-Analyses (PRISMA) 2020 statement (15).

### Search strategy

MEDLINE, EMBASE, the Clinical Trials.gov, and Cochrane Central Register of Controlled Trials (CENTRAL) has been systematically searched by two separate investigations to identify relevant studies published until May 1, 2022. The following keywords (in the title/abstract) were used: (Stereotactic body radiotherapy OR SBRT) AND (spinal metastases OR bone metastases) AND pain

### Eligibility criteria

The following are the criteria we set: (1) patient: adult patients diagnosed with painful bone metastases (a worst pain score at least ≥2 of 10, according to the Brief Pain Inventory (BPI); (2) intervention: patients who received SBRT in Intent-to-Treat Population. (3) comparison: patients who received cRT in the Intent-to-Treat Population; (4) outcomes: The primary outcome was the rate of patients with pain response at 3 months. The secondary outcomes included the rate of pain responders at 1 month and 6 months, and oral morphine equivalent dose (OMED) use. Safety outcome is adverse events. (5) study type: RCT. Included studies were not required to provide all of the aforementioned outcomes.

### Exclusion criteria

The following are the exclusion criteria we set: (1) study type: comment, letter, review, case report or case series, non-randomized; (2) language: non-English article; (3) no extractable data.

### Study selection and data collection

All entries queried from the database, RCTs, and relevant systematic reviews or meta-analyses were examined separately by the two authors (ZLW and LYL), removing duplicates and abstract-only research papers. When differences between the two authors arose, a third author (ZQC), who was not involved in the data collection procedure, made the final choices about disputed data.

### Risk of bias

The Review Manager 5.3 software was used to examine the risk of bias plot for individual research. The Cochrane Collaboration’s consistent criteria for assessing the risk of bias in RCTs were used, including selection bias, performance bias, detection bias, attrition bias, reporting bias, and other potential biases. The bias criteria were categorized as “low,” “high,” or “unclear.”

### Outcome measures

International Consensus on Palliative Radiotherapy Endpoints (ICPRE) defines a complete response (CR) as BPI = 0 with no associated increase in daily oral morphine equivalent (OME) consumption. And the partial response (PR) is defined as a reduction in the worst pain score of 2 or more points above baseline with no increase in OME, or no increase in the worst pain score and a reduction in daily OME consumption of at least 25%. Pain progression (PP) is defined as an increase from baseline in the worst pain score of 2 or more points without reduced daily OME consumption, or as no change in the worst pain score and an increase in daily OME consumption of at least 25%. Besides, any response not included in the above definition will be considered as intermediate pain (IP). CR and PR can be regarded as responders, and the others (PP and IP) are non-responders.

### Statistical analysis

The data were evaluated using STATA software 12.0 (STATA Corp., College Station, Texas, USA) by the two authors (XYY and HYT). Disputed data was solved by a third author (XXW). A random-effects model was used to assess and generate the risk ratio (RR) with the 95% confidence interval (CI) for the dichotomous outcomes. The *I^2^* statistic was used to measure heterogeneity; a value of less than 30% suggests “low heterogeneity,” a value between 30% and 50% indicates “moderate heterogeneity,” and a value of more than 50% indicates “severe heterogeneity.” The stability of the consolidated data was investigated using sensitivity analysis. Two-tailed tests were used for all of the analyses, and a *P* value of less than 0.05 was regarded as statistically significant.

## Results

### Search results and study characteristics

**Figure 1:**
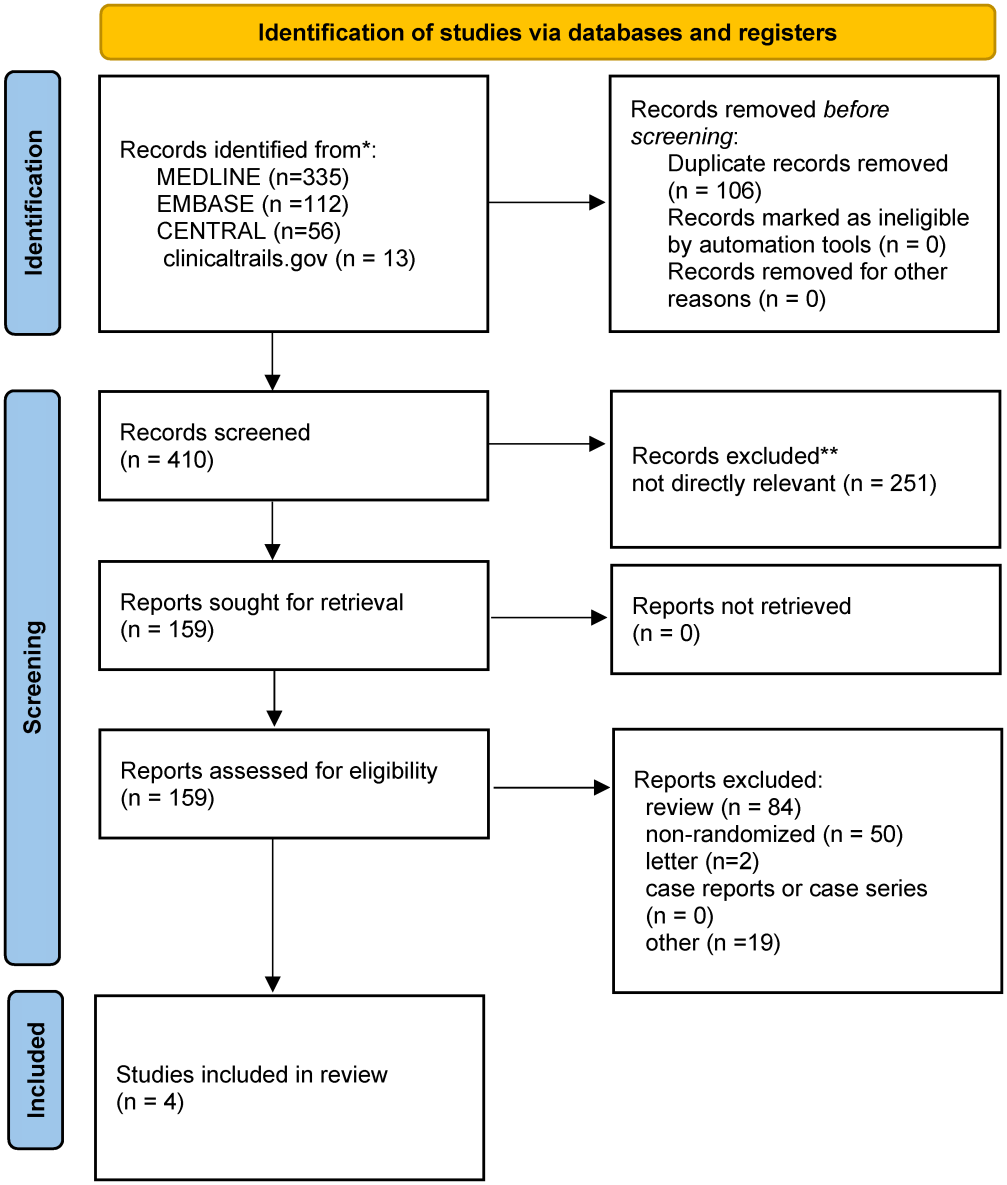
PRISMA flow diagram of study selection.

516 titles and abstracts were obtained for review from EMBASE, MEDLINE, CENTRAL, and ClinicalTrials.gov. We eliminated 106 duplicate articles and 251 irrelevant articles, leaving 159 articles, including 50 non-randomized clinical trials, 84 reviews, two letters, and 19 others. Finally, four RCTs (16–19) with 533 patients (267 in the SBRT group and 266 in the cRT group) were chosen for qualitative synthesis (**Figure 1**). **Table 1** summarizes the main characteristics of the 4 included studies. The inclusion, exclusion criteria, and outcomes of each study were shown in the supplementary materials (**Table S1**).

**Table 1:** Characteristics of the included studies and outcome events.

Study	Publications	Countries	Study design, phase, NCT	Centers	regimen	Treatment group (No. of participants)	Male (%)	age, years (IQR or Mean±SD)	Outcome events
Pielkenrood 2021 (16)	International journal of radiation oncology, biology, physics	Netherlands	RCT, phase 2, NCT02364115	1	SBRT: 18 Gy in 1 fraction, 30 Gy in 3 fractions, or 35 Gy in 7 fractions cRT: 8 Gy in 1 fraction, 20 Gy in 5 fractions, or 30 Gy in 10 fractions	SBRT: 45 cRT: 44	Surgery: 53 cRT: 70	SBRT: 65 (61-72) cRT: 63 (57-73)	a, b, d, e
Sahgal 2021 (17)	Lancet Oncology	Canada and Australian	RCT, phase 2/3, NCT02512965	18	SBRT: 24 Gy in 2 fractions cRT: 20 Gy in 5 fractions	SBRT: 114 cRT: 115	SBRT: 52 cRT: 48	SBRT: 63 (56-72) cRT: 65 (55-73)	a, b, c, d, e
Nguyen 2019 (18)	JAMA Oncology	America	RCT, phase 2, NCT02163226	1	SBRT: 12 Gy or 16 Gy in 1 fraction cRT: 30 Gy in 10 fractions	SBRT: 81 cRT: 79	SBRT: 61 cRT: 60	SBRT: 63 (52-86) cRT: 63 (23-84)	a, b, c, d, e
Sprave 2018 (19)	Radiotherapy and Oncology	Germany	RCT, phase 2, NCT02358720	1	SBRT: 24 Gy in 1 fraction cRT: 30 Gy in 10 fractions	SBRT: 27 cRT: 28	Surgery: 55.6 cRT: 46.4	SBRT: 61± 8.2 cRT: 63.9± 10.8	b, c, d, e

cRT, conventional external beam radiation therapy; IQR, interquartile range; RCT, randomized controlled trial; SBRT, Stereotactic body radiotherapy; SD, standard deviation. a: 1-mounth assessment; b: 3-mounth assessment; c: 6-mounth assessment; d: oral morphine equivalent; e: adverse events.

### Efficacy outcome

**Figure 2:**
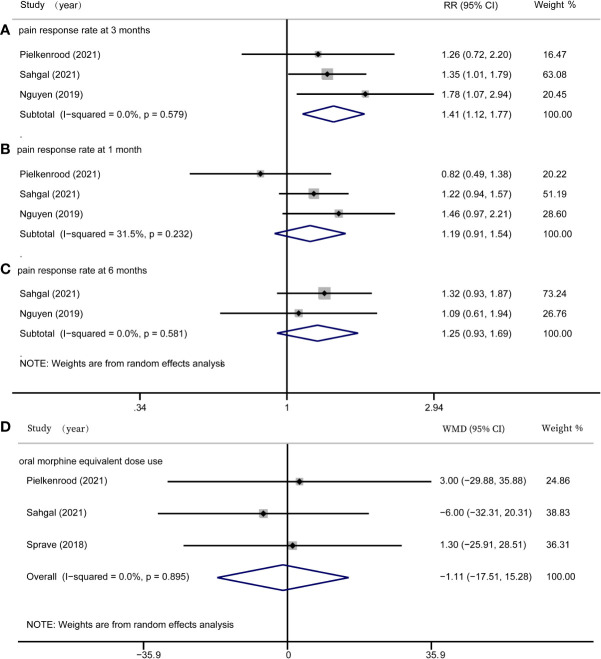
Forest plots for efficacy outcomes. **(A)** pain response rate at 3 months; **(B)** pain response rate at 1 month; **(C)** pain response rate at 6 months; **(D)** oral morphine equivalent dose use.

In this meta-analysis, we assessed rates of pain response at one month, three months, and six months after radiation, as well as OMED consumption. Three studies (16–18) reported rates of pain response at one months, three studies (16–18) reported rates of pain response at three months, and two studies (17, 18) reported rates of pain response at six months. At three months after radiotherapy, there was a significant difference of pain response rate between the SBRT group and the cRT group (RR = 1.41, 95% CI: 1.12-1.77, *I^2^* = 0.0%, P = 0.003, **Figure 2A**). However, there was no significant difference in pain response rate at one month (RR = 1.19, 95% CI: 0.91-1.54, *I^2^* = 31.5%, P = 0.201, **Figure 2B**) and six months (RR = 1.25, 95% CI: 0.93-1.69, *I^2^* = 0.0%, P = 0.140, **Figure 2C**). Three studies (16, 17, 19) investigated the OMED consumption and found it was not significantly different in patients treated with SBRT compared with controls (WMD = -1.11, 95% CI: -17.51-15.28, *I^2^* = 0.0%, P = 0.894, **Figure 2D**).

### Safety outcome

**Figure 3:**
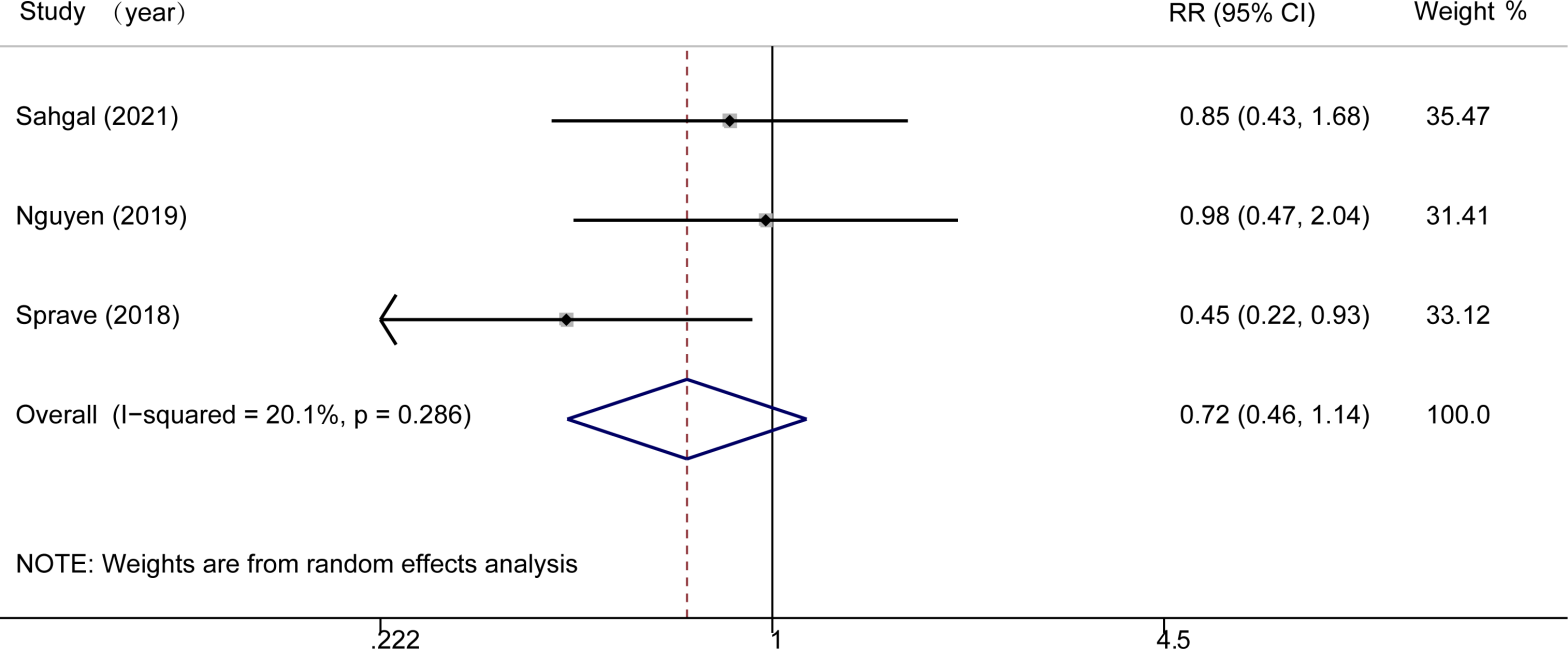
Forest plots for safety outcome.

As for the safety outcome, three out of the four studies (17–19) reported adverse events. According to our analysis, there were no statistically significant differences in adverse events such as vomiting, fatigue, or vertebral compression fracture in the SBRT group compared with the control group (RR = 0.72, 95% CI: 0.46-1.14, *I^2^* = 20.1%, P = 0.162, **Figure 3**).

### Risk of bias in included studies

**Figure 4:**
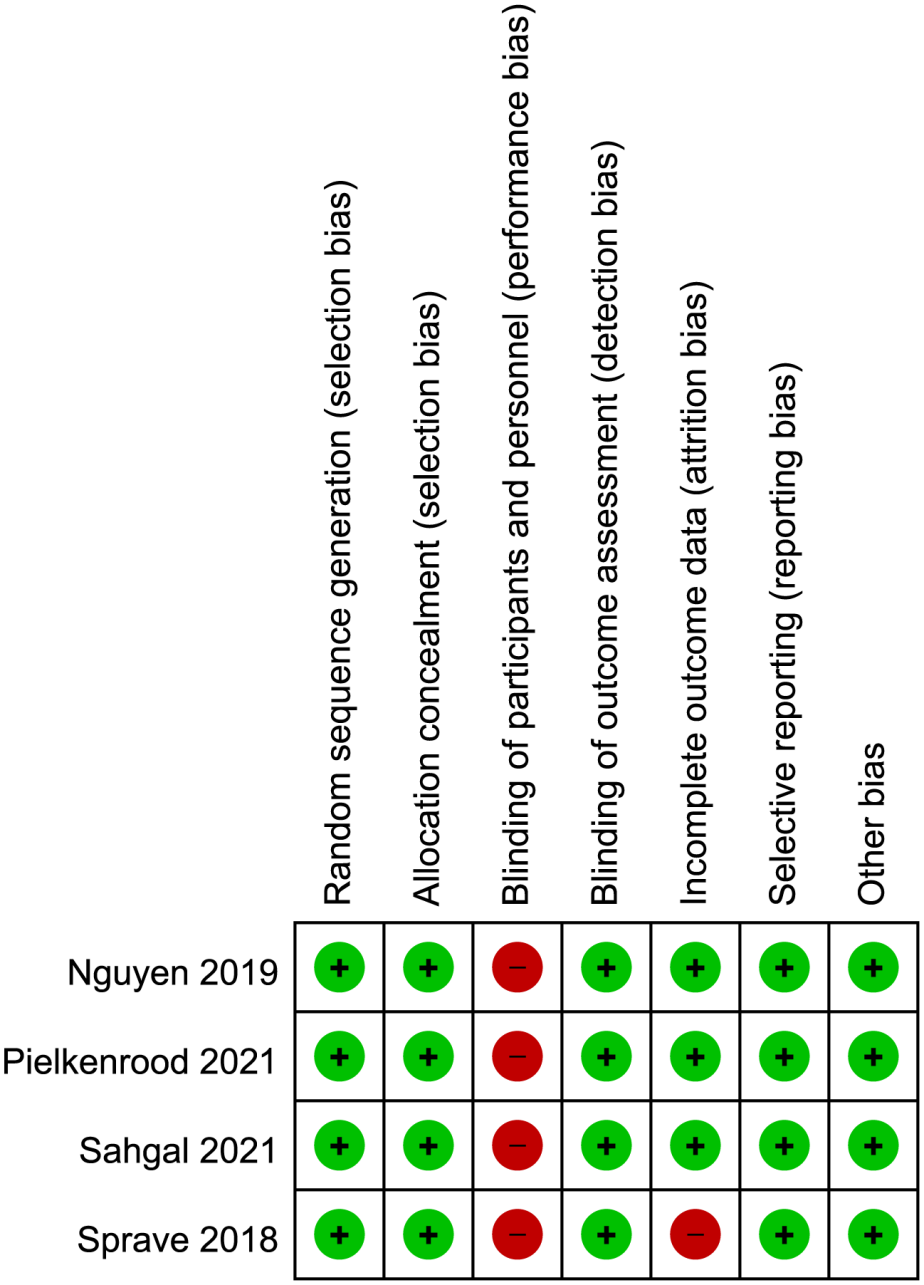
Risk of bias of the included studies.

The risk of bias for included studies were shown in **Figure 4**. All included clinical trials had a low risk of bias in random sequence generation, allocation concealment, blinding of outcome assessment, incomplete outcome data, and selective reporting. All included trials had a high risk in blinding of participants and personnel, as the differences in regimen of radiation therapy were obvious and required patient consent. The trial of Sprave et al. had a high risk in incomplete data as it only had data of the per-protocol cohort. Other items had not been found to have unclear or high risk of bias.

## Discussion

We collected four RCTs to analyze, which mainly discussed the comparison of pain relief rates between SBRT and cRT. Our meta-analysis showed that at 3 months, SBRT is significantly superior to cRT for treating painful bone metastases pain. However, no significant difference has been found between SBRT and cRT group in the pain response at one month and six months. In addition, compared to the control group, SBRT group failed to reduce the use of OMED significantly.

Overall, according to our analysis, only pain response at 3 month was significantly improved in SBRT group. Several factors may attribute to the negative results at 1 month and 6 months between SBRT group and cRT group. Firstly, for pain response at 1 month, both Pelkenrood’s trial and Sahgal’s trial concluded that SBRT did not significantly improve pain response, while Nguyen’s trial drew the opposite conclusion. For pain response at 6 months, only Sahgal’s trial and Nguyen’s trial were included. As Nguyen’s trial mainly included patients with nonespinal metastases, it may interfere with the results’ extrapolation to the general population with spinal metastases. When evaluating which patients should be treated with SBRT, those RCTs also offered conflicting opinions. Sahgal et al. and Sparve et al. proposed that SBRT provides faster and better pain relief and should be recommended for patients with shorter expected survival (17, 19). Nguyen et al. proposed that SBRT has a persistent pain response rate, it should be the standard of care for patients with longer life expectancy and fewer bone metastases (18). Despite this disagreement, they agreed that SBRT was a better regimen for painful bone metastasis. Though no significant difference has been found for pain response at 1 month and 6 months, SBRT group tends to have a better efficacy outcome. Taking the above points into consideration, we hold the opinion that the application of SBRT to relief pain in patients with bone metastases of cancer has an anticipated clinical application prospect.

Radiation therapy has been considered as the main treatment for cancer with bone metastases. Radiation therapy uses externally generated electromagnetic and ion beams fired into the body to induce interactions between chemical free radicals and DNA, or other intracellular targets, leading to apoptosis (20). For conventional external radiation, there are two treatment options: single-fraction and multi-fraction radiotherapy, both has been widely used in clinical practice. Single-fraction is 8Gy in 1 fraction, multi-fraction adopts several regimens such as 30Gy in 10 fractions, 15Gy in 5 fractions, 20Gy in 5 fractions. Debate continues over which fractionation regimen is the best treatment for painful bone metastases. However, past studies have shown no significant difference in pain relief rates between single-fraction and multi-fraction (21–23).

In the past ten years, there has been a revolution in radiation therapy, SBRT is a novel treatment for cancer with spinal and bone metastases, as it can deliver higher doses precisely to the cancer cells in the target area (8). In 2012, the International Spinal Radiosurgery Consortium published a new standard for determining target volume of SBRT (24). Multiple prospective studies showed that SBRT could better control spinal metastasis tumors (9, 25). Thus, SBRT has been considered as a new treatment option that can bring benefits to patients. As for pain relief, one of the primary endpoints for palliative radiotherapy, a study by Owen et al. showed that the pain relief rate of SBRT in the treatment of non-spinal bone metastasis reached 88% (10), while Wang et al. and Chang et al. reported that the pain relief rate of SBRT in the treatment of spinal bone metastasis reached 50% (11, 26).

Studies with a higher level of evidence are urgently required to verify the efficacy and safety of SBRT for pain relief. In 2019, a prospective study compared SBRT with cRT plan, they put forward that SBRT can improve patients’ quality of life (27). Sakr et al. proposed that SBRT had better immediate pain relief than cRT (28). Results of an RCT in 2018 showed that palliative SBRT treatment for spinal metastatic tumors had a faster and better pain response rate (19). Subsequently, Sah et al. proposed that SBRT is superior to cRT for symptom control in patients with less metastatic disease (17). Based on these views, we conducted a meta-analysis to compare the pain relief rates between the two regimens. Considering the previous views and the result of our analysis, the effectiveness of SBRT in relieving pain of bone metastases might be promising, especially at three months after treatment.

In addition, in terms of the safety of the two regimens, three of the four RCTs we included reported adverse events, including dysphagia, oesophagitis, nausea, pain, fatigue, radiodermatitis, and vertebral compression fracture. No statistical difference was found according to our meta-analysis, indicating the safety of SBRT as compared to cRT. However, the probability of spinal compression fractures after SBRT should not be ignored, a systematic review by Sahgal et al. analyzed risk factors for pathologic fracture after SBRT, and they put forward that the patients with SBRT have a higher risk of pathological fracture (29). To note, the RCT conducted by Sahgal et al. demonstrated that more patients in the cRT group had a vertebral compression fracture than SBRT group. Overall, despite the risk of pathological fractures and other adverse events exist, the safety of SBRT is still promising.

There are some limitations in this meta-analysis. Firstly, our analysis was based on limited data from four published RCTs to test the efficacy and safety of SBRT. And only three out of the four RCTs were included for the analysis of our primary outcome as Sprave et al.’s study did not reported data of the Intent-to-Treat analysis. Secondly, different extracorporeal radiotherapy segmentation schemes were adopted in the included RCTs. Thirdly, the study by Pelkenrood et al. only reported follow-up data within 3 months. Lastly, the response to SBRT and subsequent pain response may be impacted by the variations in tumor histology and molecular subtype. These are all possible reasons for heterogeneity.

In the future, defining the most effective and safe SBRT dose and segmentation schemes are new directions for SBRT development. Further larger randomized controlled trials are still required to determine the optimum treatment for patients with painful bone metastases.

## Conclusion

In conclusion, this study shows that for painful bone metastases, patients with SBRT experienced better pain relief three months after radiation than patients with cRT, and did not increase the incidence of adverse events. For patients who expect better quality of life after palliative radiotherapy, SBRT seems to have a promising application prospect.

## Data availability statement

The original contributions presented in the study are included in the article/**Supplementary Material**. Further inquiries can be directed to the corresponding authors.

## Author contributions

ZLW and LL was the principal investigator. ZLW, LL and ZC designed the study and developed the analysis plan. XY, HT and XW analyzed the data and performed meta-analysis. ZLW and LL contributed in writing of the article. XY and HT revised the manuscript and polish the language. ZC, ZW and GC supervised the project. All authors contributed to the article and approved the submitted version.

## Funding

This work was supported by the Natural Science Foundation of Jiangsu Province (Grants No. BK20200203) and the National Natural Science Foundation of China (Grant No. 81873741).

## Acknowledgments

We appreciate the valuable and constructive suggestions and assistance from our Team of Neurosurgical study.

## Conflict of interest

The authors declare that the research was conducted in the absence of any commercial or financial relationships that could be construed as a potential conflict of interest.

## Publisher’s note

All claims expressed in this article are solely those of the authors and do not necessarily represent those of their affiliated organizations, or those of the publisher, the editors and the reviewers. Any product that may be evaluated in this article, or claim that may be made by its manufacturer, is not guaranteed or endorsed by the publisher.
