# Supplementary material for: Efficacy and safety of stereotactic body radiotherapy for painful bone metastases: Evidence from randomized controlled trials
Source: Front Oncol. 2022 Oct 19;12:979201. doi: 10.3389/fonc.2022.979201 (PMC9627033; doi:10.3389/fonc.2022.979201)
Supplement: Supplementary file 1 [file Table_1.docx]

| **Table S1: Inclusion, exclusion criteria, and outcome assessments of the included studies** | | | | |
| --- | --- | --- | --- | --- |
| Studies | Inclusion Criteria | Exclusion Criteria | Efficacy Outcomes | Safety Outcomes |
| Pielkenrood 2021^16^ | KPS score >50 points, and pain score ≥3. Histologic proof of malignancy, radiologic or histologic evidence of bone metastases, no more than 2 painful lesions requiring treatment, no compression of spinal cord/cauda equina, no or mild neurologic signs such as (radiating) pain or numbness, | Contraindications to undergo MRI, metastasis from a highly radiosensitive tumor (eg, lymphoma), lesions too large for SBRT (ie, >10 cm), estimated life expectancy less than 3 months, previous cRT or SBRT on the same level, need for surgical stabilization, and severe, worsening, or progressive neurologic deficits (eg, muscle weakness). | 2-weeks assessment, 4-week assessment, 6-week assessment, 8-week assessment, 3-month assessment of pain response, daily oral morphine equivalent | Global QOL scores of the EORTC-QLQC15 questionnaire. |
| Sahgal 2021^17^ | Aged 18 years or older with painful (defined as a worst pain score of ≥2 of 10, according to the BPI; MRI-confirmed spinal metastases who had no intention of changing pain medications on the first day of protocol radiotherapy treatment, had no more than three consecutive spinal segments in the radiotherapy treatment volume site, had an Eastern Cooperative Oncology Group performance status of 0–2, had metastases arising from a solid primary tumour (excluding seminoma and small-cell lung cancer), had a Spinal Instability in Neoplasia Score of 12 or less, had received no previous radiotherapy that would compromise the study interventions, had undergone no previous spinal surgical procedures at the study target volume site, and had no neurological deficits resulting from malignant epidural spinal cord or cauda equina compression. Systemic chemotherapy was not allowed at least 1 week before and after study radiotherapy delivery, and centre guidelines applied with respect to non-cytotoxic systemic therapy, with the proviso that no systemic anticancer therapy (excluding endocrine therapy) be administered within 24 h before or after radiotherapy. | - | 1-month assessment, 3-month assessment, 6-month assessment of pain response, daily oral morphine equivalent, change in the total Spinal Instability in Neoplasia Score from baseline at 3 and 6 months. | RTQA compliance, radiation site-specific progression-free survival, and QOL score and adverse events. |
| Nguyen 2019^18^ | Age ≥ 18 years with a life expectancy of more than 3 months. Pathologic diagnosis of cancer, painful bone metastases (ie, a score of at least 2 on a 0-to-10 scale), Concurrent treatment of up to 3 radiation fields was allowed. | Prior radiation to the site being evaluated, untreated spinal cord compression, pathologic fracture at the evaluated site, and previous receipt of radioactive isotope therapy (eg, strontium 89) within 30 days of randomization. | 2-weeks assessment, 1-month assessment, 3-month assessment, 6-month assessment, 9-month assessment of pain response. | Overall survival estimates, MD Anderson Symptom Inventory score, toxic effects. |
| Sprave 2018^19^ | Ages 18–80, KPS ≥70, ability to provide written informed consent, a maximum of two irradiated vertebral bodies per region, a maximum of two different vertebral regions affected, and tumor distance >3 mm to the spinal cord. | Subjects with significant neurological or psychiatric disorders precluding informed consent, previous RT to the given irradiation site, contraindications for MRI, multiple myeloma or lymphoma histology, or involvement of the cervical spine. | 3-month assessment, 6-month assessment of pain response, daily oral morphine equivalent. | Overall survival, bone survival and adverse events. |
| BPI, Brief Pain Inventory; cRT, conventional external beam radiation therapy; KPS, Karnofsky performance status; QOL, quality of life; RT, radiation therapy; SBRT, stereotactic body radiotherapy | | | | |
